# Global Research Trends and Hotspots in Cerebral Small Vessel Disease‐Related Cognitive Impairment: A Bibliometric Analysis (2001–2024)

**DOI:** 10.1002/brb3.71167

**Published:** 2026-01-25

**Authors:** Kuihua Wang, Xiaohui Ji, Hui Li, Xiaoyang Wang, Xiaoyue Jin, YanJun Lin, Qiao Wang, Haizhen Xu, Jianxin Ye, Xiaoping Cui, Yonghui Liang

**Affiliations:** ^1^ Department of Neurology 900th Hospital of PLA Joint Logistic Support Force Fuzhou China; ^2^ Department of Neurology Fuzong Clinical Medical College of Fujian Medical University Fuzhou China; ^3^ Department of Neurology Fuzong Teaching Hospital of Fujian University of Traditional Chinese Medicine (900th Hospital) Fuzhou China; ^4^ Department of Otolaryngology The Third People's Hospital Affiliated to Fujian University of Traditional Chinese Medicine Fuzhou China; ^5^ Fujian Psychiatric Center Fujian Clinical Research Center for Mental Disorders Department of Medical Imaging Xiamen Xianyue Hospital, Xianyue Hospital Affiliated with Xiamen Medical College Xiamen China

**Keywords:** bibliometric analysis, cognitive impairment, CSVD, research trends

## Abstract

**Background and aims:**

Cerebral small vessel disease (CSVD) is a significant contributor to cognitive impairment (CI). Research on CSVD‐CI has gained prominence; however, comprehensive bibliometric analysis of the global research landscape in this area is currently lacking. This study aims to address this gap by conducting a thorough bibliometric analysis to identify research hotspots and emerging trends.

**Methods:**

We retrieved relevant literature published from 2001 to 2024 from the web of science (WoS) core collection. using VOSviewer, CiteSpace, R, and Origin 2022, we performed bibliometric and network visualization analyses to examine publication trends, geographical distribution, institutional collaborations, journal metrics, citation patterns, author contributions, co‐citation networks, and keyword co‐occurrence.

**Results:**

Three distinct phases were identified: foundational (2001–2010), developmental (2011–2018), and accelerated (2019–2024). The United States exhibited the highest citation impact, whereas China reported the highest number of publications. The journals “neurology” and “stroke” emerged as the leading publications. Four major research clusters were revealed: pathophysiological mechanisms (neuroinflammation and oxidative stress), neuroimaging biomarkers (white matter hyperintensities (WMH) and cerebral micro bleeds), clinical manifestations and cognitive assessment, and therapeutic interventions. Emerging trends included blood‐brain barrier (BBB) dysfunction, neurovascular coupling, and innovative therapies.

**Conclusions:**

This bibliometric study sheds light on the research hotspots and trends in CSVD‐CI over the past two decades, assisting researchers in identifying key focus areas and exploring advancements in this domain.

## Graphical abstract

This bibliometric study systematically analyzed 1074 publications in the field of CSVD‐CI from 2001 to 2024. The research reveals three evolutionary phases and identifies four core research clusters: pathophysiological mechanisms, neuroimaging biomarkers, clinical assessment, and therapeutic interventions. China contributed the highest number of publications, while the Netherlands demonstrated the strongest academic influence. The analysis clarifies the evolution of research hotspots and international collaboration patterns, offering comprehensive guidance for future research in this field.

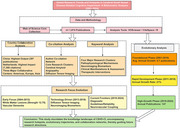

## 1 Introduction

CSVD is a prevalent cerebrovascular disorder that primarily affects the small arteries, arterioles, capillaries, and venules in the brain (Wardlaw et al. 2013). It is not only a major cause of stroke but is also strongly associated with various neurological dysfunctions, including CI, dementia, gait disturbances, and mood disorders (Rensma, van Sloten, Launer, and Stehouwer 2018). In recent years, against the backdrop of a rapidly aging global population, the incidence of CSVD and its related CI (CSVD‐Related CI, CSVD‐CI) has been rising steadily, emerging as a significant public health challenge worldwide.

The pathological mechanisms underlying CSVD‐CI are complex and involve multiple processes, including vascular endothelial dysfunction, BBB disruption, chronic cerebral ischemia, white matter lesions (WMLs), and microhemorrhages (Hannawi 2024; Kern et al. 2024). These pathological changes ultimately lead to neuronal damage and synaptic dysfunction, resulting in cognitive decline (Kern et al. 2024).

Advancements in scientific technology and diversified research methodologies have spurred a growing volume of global research on CSVD‐CI, spanning from basic science to clinical applications. However, a comprehensive and systematic analysis of the research landscape in this field is currently lacking. In particular, there is an absence of studies from a bibliometric perspective to explore research hotspots, developmental trends, and collaborative networks.

As a quantitative method for analyzing scientific literature, bibliometrics can reveal a field's research status, developmental trajectory, and knowledge structure by statistically analyzing publications' volume, authors, institutions, countries, and keywords, thereby providing valuable insights for researchers (Wang et al. 2024; Ye et al. 2024).

This study aims to conduct a systematic bibliometric analysis of CSVD‐CI‐related literature published between 2001 and 2024, comprehensively revealing the global research landscape, key research forces, collaborative networks, research hotspots, and frontier trends in this field. Through this analysis, we hope to provide a scientific basis for future research directions, promote international collaboration, and advance the further development of CSVD‐CI research, ultimately contributing to improved patient quality of life and alleviated societal burdens.

The significance of this study is threefold. First, it employs bibliometric methods to systematically delineate the current research landscape of CSVD‐CI, thereby addressing a gap in comprehensive analysis. Second, it identifies research hotspots and emerging trends, providing valuable direction for researchers. Finally, by mapping international collaborative networks, it aims to foster global academic exchange and cooperation, thereby promoting interdisciplinary integration and innovation in CSVD‐CI research.

## 2 Methods

### 2.1 Study Design and Registration

This study is a retrospective bibliometric analysis that exclusively uses data from published scientific literature. As no clinical trial was conducted, and no human participants or animals were involved, this study does not require a clinical trial registration number in accordance with ICMJE guidelines.

### 2.2 Ethics Statement

This study is a bibliometric analysis based on data extracted from the WoS core collection, a publicly available scientific citation database. As the research exclusively involves the analysis of previously published literature and does not include any direct involvement of human participants, animal subjects, or collection of primary personal data, it did not require review or approval by an institutional ethics committee. All data were handled in accordance with the terms and conditions of the database provider. The authors declare no conflicts of interest related to this work.

### 2.3 Data Acquisition

The WoS database served as the primary data source for this bibliometric analysis. Renowned for its comprehensive and multidisciplinary citation data, it enables researchers to access and collect extensive bibliographic information from numerous prestigious journals (Wang et al. 2024; Ye et al. 2024).

Our systematic search strategy was implemented as follows: (1) the topic search (TS) was conducted using the following query: “((”CSVD*“) or (”cerebral small vascular disease*“) or (”cerebral microvascular disease*“) or (”cerebral small blood vessel disease*“)) and ((”cogni* disorder*“) or (”cogni* impairment*“) or (”cogni* deficit*“) or (”cogni* dysfunction“) or (”cogni* barrier*“))”; (2) the search encompassed all available years without temporal restrictions; (3) only English‐language publications were included; and (4) Exclusion of reviews, conference papers, and other non‐relevant types, yielding a final set of 1074 pertinent publications.

The final literature search was conducted on December 10, 2024. This specific date was selected to establish a consistent year‐end cut‐off point. According to bibliometric guidelines, employing a fixed data retrieval point is crucial for ensuring the comparability and replicability of annual trend analyses (Donthu et al. 2021). Thus, this practice of setting a year‐end cut‐off has become a common analytical standard in the field (Öztürk et al. 2024).

The literature search and screening process were performed independently by two authors (Kuihua Wang and Xiaohui Ji). Any discrepancies regarding the pertinence of articles or document type classification were resolved through discussion until a consensus was reached.

All relevant documents and their complete metadata (including titles, authors, abstracts, keywords, and citation data) were exported from the WoS database in plain text format, providing a robust foundation for subsequent bibliometric analysis.

The complete list of the 1074 references analyzed in this study is available in Supplementary File 1.

### 2.4 Analysis

As a set of essential methods for the quantitative assessment of academic literature, bibliometric analysis leverages tools such as VOSviewer (van Eck and Waltman 2010), CiteSpace (Chen 2017), and the Bibliometrix R‐package (Aria and Cuccurullo 2017) to facilitate the visualization and statistical analysis of publication data.

VOSviewer specializes in constructing and visualizing bibliometric networks, including those of citation, bibliographic coupling, co‐citation, and co‐authorship (Arruda et al. 2022; van Eck and Waltman 2010). It also enables text mining for building and visualizing co‐occurrence networks of key terms extracted from large bodies of scientific literature (Chen 2017).

CiteSpace is a versatile tool for identifying emerging trends and pivotal developments in scientific literature (Chen 2017). It performs both structural and temporal analyses of bibliographic data from the WoS, generating diverse networks such as collaboration, author co‐citation, and bibliographic coupling. The tool is widely recognized for its capabilities in burst detection and time‐zone visualization, which aid in mapping research trajectories and anticipating future directions (Chen 2006, 2017).

Bibliometrix, an R‐based package, offers a comprehensive suite for bibliometric analysis, encompassing the entire process from data import and transformation to analysis and visualization. Its robust functionalities include the generation of bibliographic coupling, co‐citation, co‐authorship, and co‐occurrence networks (Aria and Cuccurullo 2017)

In this study, we employed Bibliometrix (v4.3.1), CiteSpace (v6.2.R7), and VOSviewer (v1.6.20) to conduct a comprehensive bibliometric analysis of CSVD‐CI research. Each tool was selected for its distinct capabilities, which together provide complementary insights into the research landscape.

All tables (Tables 1 through 5) have been provided as editable worksheets within a single supplementary Excel file (BRB3‐2025‐05‐1140_R3_Tables.xlsx).

## 3 Results

### 3.1 Annual Publication Trend

**FIGURE 1:**
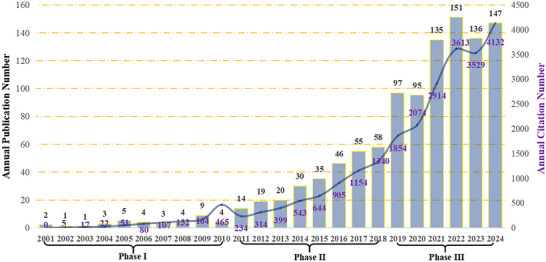
Historical trends in annual publications and cumulative citations on CSVD‐CI research (2001–2024). The bar chart depicts the annual number of publications (left Y‐axis). The line graph shows the cumulative citation count over time (right Y‐axis). The background is shaded to indicate the three developmental phases identified in this study: Foundational (2001–2010, light blue), developmental (2011–2018, light orange), and accelerated (2019–2024, light gray).

The annual publication output from 2001 to 2024 reveals a pronounced non‐linear growth pattern, which we categorized into three distinct phases based on the growth slope (i.e., the rate of change in the number of annual publications) and the underlying research drivers (Figure 1).

Phase 1: Foundational and incremental Growth (2001–2010)

This period was marked by a slow growth rate, with the annual number of publications rising from 2 to 9, resulting in an average of only about 0.7 papers per year. This slow progress indicates that the field was still in its infancy, with research primarily focused on establishing the fundamental clinical and pathological links between CSVD and CI. The limited output suggests that the concept of CSVD‐CI was still being defined and was gradually gaining recognition within the broader research community.

Phase 2: Accelerated expansion (2011–2018)

This phase was defined by a marked increase in the growth rate, with annual publications surging from 14 to 58, representing a compound annual growth rate (CAGR) of approximately 21%. This acceleration can be attributed to two key factors: (1) the widespread adoption and standardization of advanced neuroimaging techniques (e.g., DTI and SWI), which enabled more precise in vivo quantification of CSVD burden; and (2) the publication of influential consensus documents, such as the STRIVE‐1 criteria in 2013 (Wardlaw et al. 2013), which established a common nomenclature for researchers and stimulated large‐scale, multi‐center collaborative studies.

Phase 3: High‐velocity growth (2019–2024)

The field entered a phase of sustained, rapid expansion, with annual publications increasing from 97 to 147. Although this phase recorded the highest absolute number of new publications annually, the CAGR exhibited relative stabilization or a slight decline compared to Phase 2. This trend signals a transition toward a more linear growth pattern as the field matured. The period is characterized by two key developments: a diversification of research themes—with deeper investigations into pathophysiological mechanisms such as neuroinflammation and BBB dysfunction—and a growing emphasis on translating basic research discoveries into potential therapeutic and preventive strategies.

In conclusion, the period from 2001 to 2024 was marked by sustained expansion in both publication output and citation impact within the field of CSVD‐CI research. The field's evolution transitioned from establishing foundational knowledge to a multifaceted exploration of pathological mechanisms, neuroimaging biomarkers, clinical manifestations, and therapeutic interventions.

### 3.2 Analysis of Journals and Co‐Cited Journals

A total of 325 academic journals published articles on CSVD‐CI. As shown in Table 1, the top 10 journals accounted for one‐third of all publications. Stroke (impact factor: 4.76) was the most productive journal, with 56 articles (5.21%), followed by frontiers in aging neuroscience and the journal of alzheimer's disease. The top five journals each published more than 40 articles, representing over 3.5% of total publications, thereby establishing their prominence in CSVD‐CI research. Among these top 10 journals, the average Impact Factor was 5.73, with four journals having an Impact Factor above 5.0, which underscores the growing academic importance of this research field.

**TABLE 1:** Ranking of top 10 journals and co‐cited journals involved in the CSVD‐CI domain.

Publication titles	Record count	% of 1074	IF	Cited journal	Record counts
Stroke	56	5.21%	4.76	Neurology	936
Frontiers in aging neuroscience	50	4.66%	4.1	Stroke	920
Journal of alzheimers disease	47	4.38%	3.4	Lancet neurol	875
Frontiers in neurology	44	4.10%	2.7	Brain	548
Neurology	40	3.72%	9.9	J neurol neurosur Ps	543
Neuroimage‐clinical	29	2.70%	4.89	J alzheimers dis	440
Alzheimers and dementia	26	2.42%	13	Neuroimage	372
European journal of neurology	20	1.86%	5.1	Alzheimers dement	359
Scientific reports	16	1.49%	4.44	J cerebr blood F met	348
Alzheimers research and therapy	15	1.40%	5.02	Plos one	321
Human brain mapping	15	1.40%	4.8	Neurobiol aging	321
Journal of stroke and cerebrovascular diseases	15	1.40%	2.02		

*Note*: 1. Data source: Web of Science Core Collection (2001–2024), with 1,074 CSVD‐CI‐related publications included (see Methods section). 2. Ranking basis: Journals ranked by the number of CSVD‐CI publications; proportion calculated as: (journal's publication count / 1074) × 100%. 3. Citation metrics:'Total Citations' refers to citations of included CSVD‐CI articles; 2023 Journal Impact Factor and JCR Quartile are annual reference values. 4. Interdisciplinary feature: Journal distribution reflects the interdisciplinary integration of cerebrovascular, neurodegenerative, neuroimaging, and gerontological research in the CSVD‐CI field.

**FIGURE 2:**
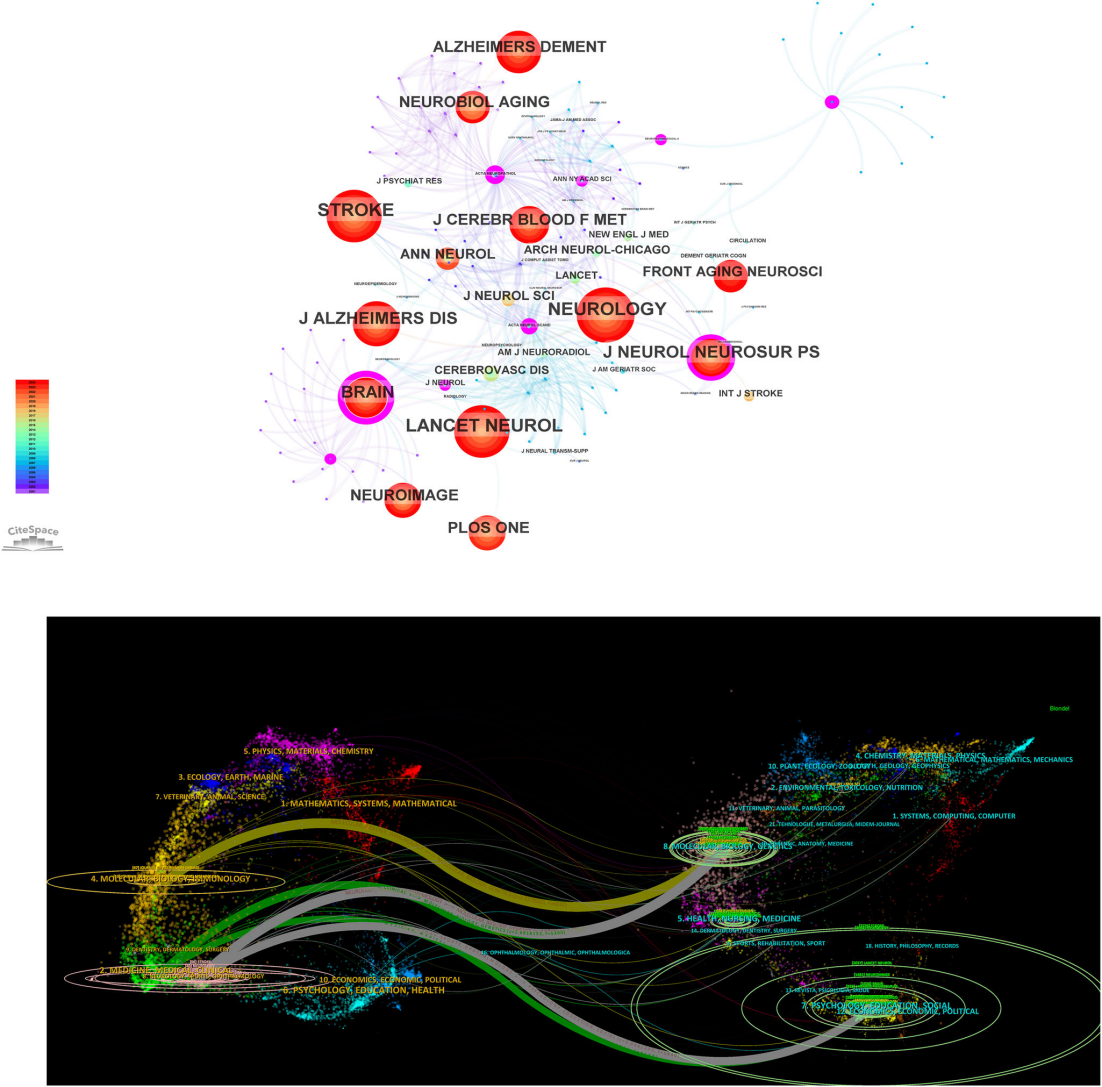
Journal analysis in CSVD‐CI research. (A) Co‐citation network of journals. Nodes represent journals, and their size is proportional to the co‐citation frequency. Lines between nodes represent co‐citation relationships; and (B) dual‐map overlay of journals. The left side shows citing journals, and the right side shows cited journals. Curved lines represent the main citation pathways between disciplines.

In the journal co‐citation analysis, neurology ranked first with 936 citations, followed by stroke (920 citations) and lancet neurology (875 citations). These high citation frequencies highlight the academic influence of these periodicals and their established role as authoritative sources in CSVD‐CI research. The journal co‐citation network, generated using CiteSpace, consisted of 141 nodes and 504 links (Figure 2A). These journals collectively focus on investigating the relationship between CSVD and CI, exploring their implications for neurological practice.

The scope of these publications spans a broad spectrum from basic science to clinical applications, covering cerebrovascular disorders, alzheimer's disease, dementia, advances in neuroimaging, and neurobiological changes related to aging. This research aims to advance diagnostic methods, therapeutic interventions, and preventive strategies. Since cited journals provide the foundational knowledge for citing publications, the observed diversity reflects an evolving scholarly focus from single‐discipline approaches to multidisciplinary research clusters.

The dual‐map overlay of journals (Figure 2B) illustrates the citation pathways within CSVD‐CI literature, with citing journal categories on the left and cited journal disciplines on the right. Five major citation trajectories were identified: (1) the light orange pathway indicates that journals in “molecular, biology, and immunology” predominantly cited research from “molecular, biology, and genetics”; (2) the gray pathway shows citations from “neurology, sports, and ophthalmology” journals to both “molecular, biology, and genetics” and “health, education, and psychology”; and (3) the green pathway reveals that “medicine, medical, and clinical” journals primarily referenced “molecular, biology, and genetics” and “health, education, and psychology”. Collectively, these pathways demonstrate an interdisciplinary research approach that integrates molecular biology with psychosocial perspectives to address complex neurological disorders.

### 3.3 Collaborative Networks

#### 3.3.1 International Collaboration Among Countries/Regions

**FIGURE 3:**
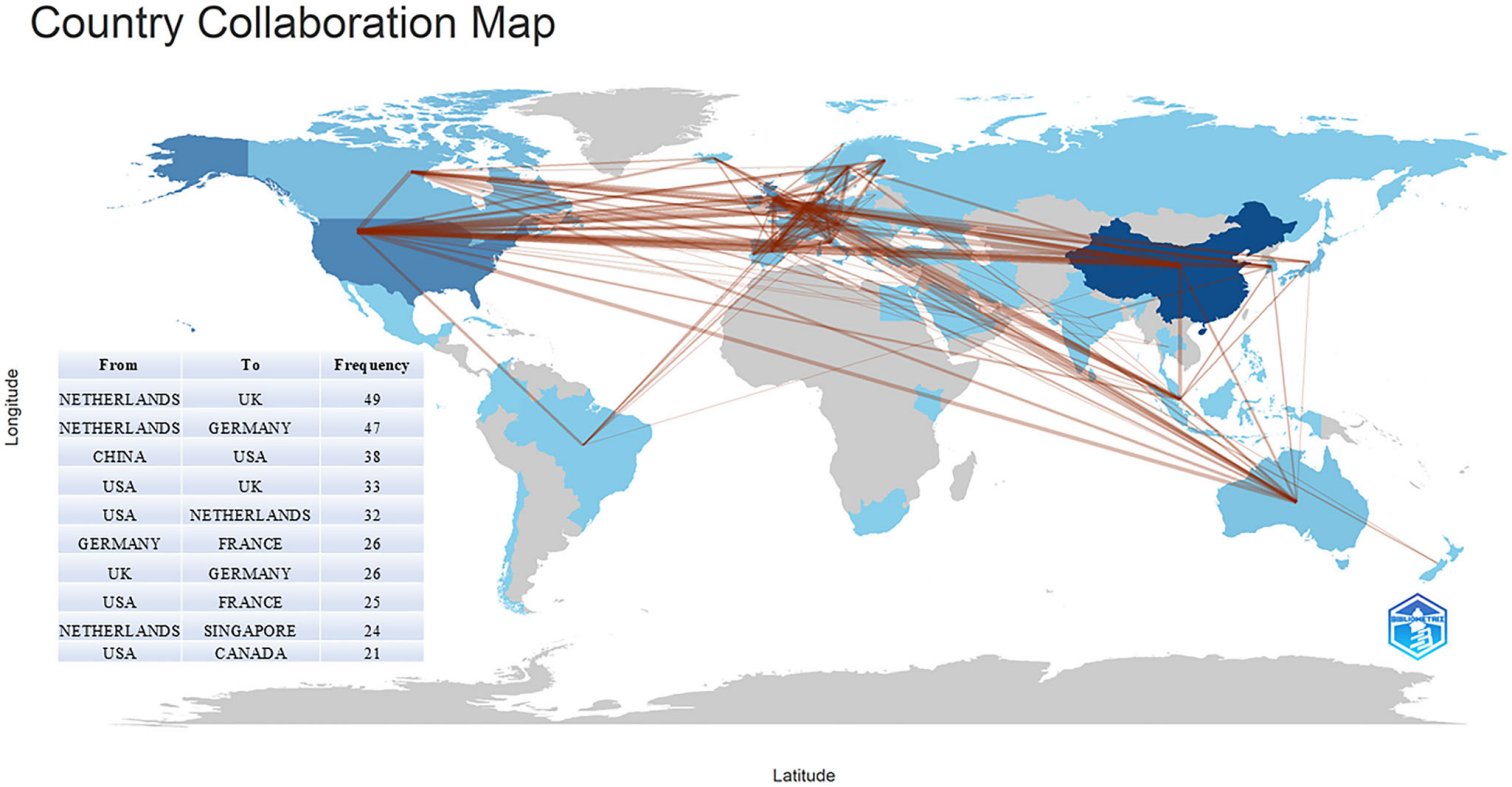
Geographical distribution and international collaboration in CSVD‐CI research. The map visualizes the number of publications from different countries/regions (represented by color intensity) and their collaborative relationships (represented by connecting lines).

Figure 3 presents a comprehensive visualization of international collaboration in CSVD‐CI research. The network map depicts interconnections between countries/regions, with link thickness proportional to the collaboration strength (Donthu et al. 2021).

Analysis of the top ten countries reveals the strongest bilateral collaboration between the Netherlands and the United Kingdom (49 publications), followed by the Netherlands and Germany (47 publications). Other major partnerships each produced more than 20 collaborative publications. The United States maintains the most extensive network, with balanced collaboration across partners. China, France, and Singapore have also established robust international networks.

The global landscape of CSVD‐CI research includes 57 countries/regions, though geographical disparities are apparent, with notably lower participation from West Asia, South Asia, Africa, Eastern Europe, and southern South America. China leads in publication output (397 publications), followed by the United States (222) and the Netherlands (177). Regarding citation impact, the Netherlands leads with 7300 citations, followed by the UK (6323) and the United States (6120), indicating varying regional influence.

**FIGURE 4:**
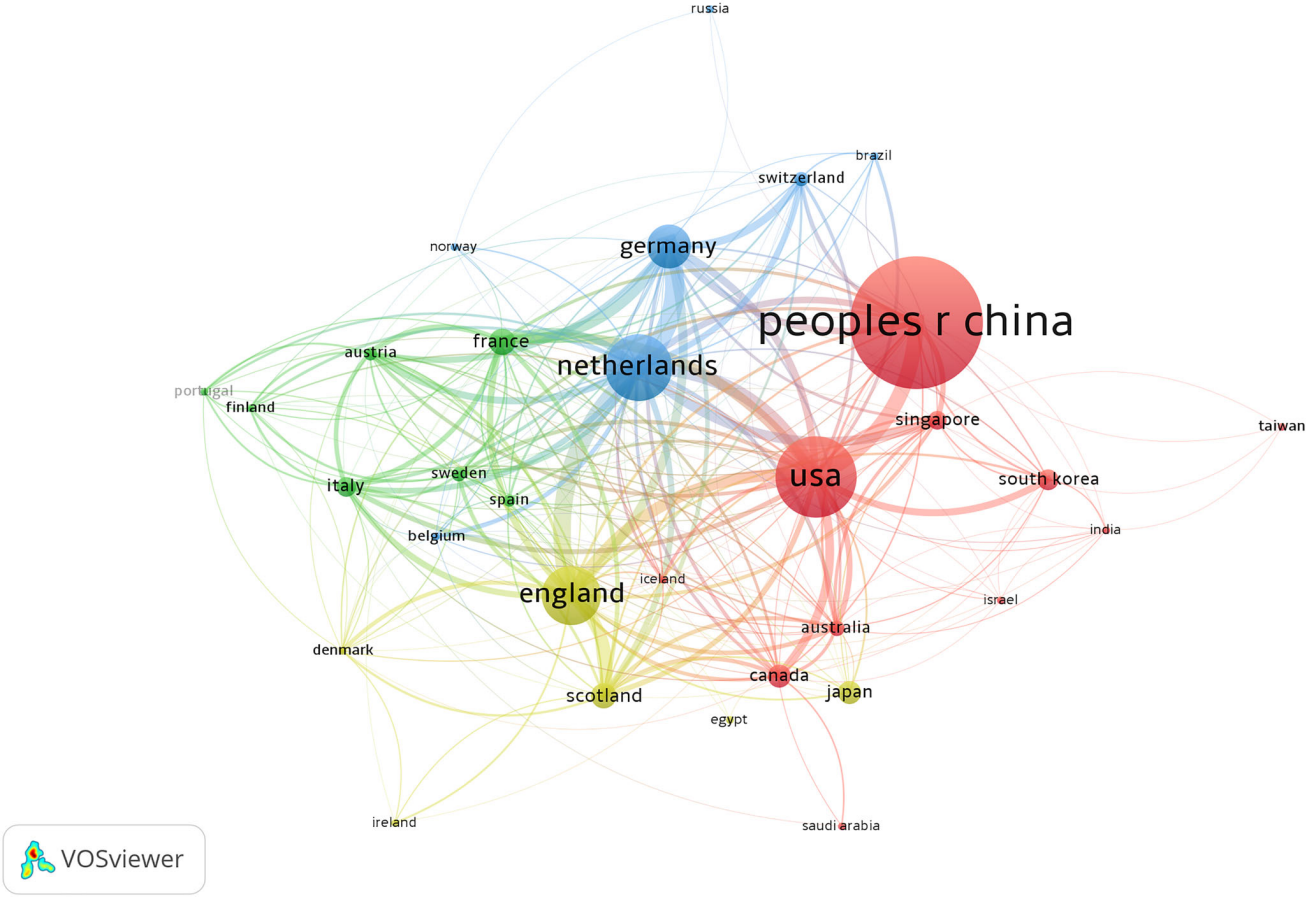
Network visualization of international collaboration among countries/regions. This analysis included 31 countries/regions that met the threshold of a minimum of five publications and had collaborative links. Nodes represent countries/regions, with their size proportional to publication output and colors indicating distinct collaboration clusters identified by VOSviewer's clustering algorithm. Links between nodes indicate collaborative relationships, with thickness representing the strength of bilateral collaboration.

Setting a minimum threshold of five publications per country/region and excluding non‐collaborating entities, 31 countries/regions were incorporated into the network analysis. These were classified into four distinct collaboration clusters (Figure 4) using VOSviewer's clustering algorithm (van Eck and Waltman 2010) for network mapping (Donthu et al. 2021). The network reveals China, the United States, the Netherlands, the UK, and Germany as the most central and interconnected entities. Notably, despite their strong interconnectivity, these five nations are distributed across different clusters, a pattern suggesting that geographical proximity continues to influence research focus and collaborative dynamics in CSVD‐CI.

The analysis identifies China, the United States, the Netherlands, the UK, and Germany as the five most prominent nodes, with substantial sizes and thick connecting links indicating strong collaborative ties. Notably, although strongly interconnected, these five countries are distributed across different clusters (shown by distinct colors). This clustering pattern suggests that geographical proximity remains an influential factor in shaping research focus and collaboration in CSVD‐CI. Enhanced cross‐cluster collaboration could accelerate scientific progress in understanding CSVD‐related CI.

#### 3.3.2 Institution Cooperation Network

**FIGURE 5:**
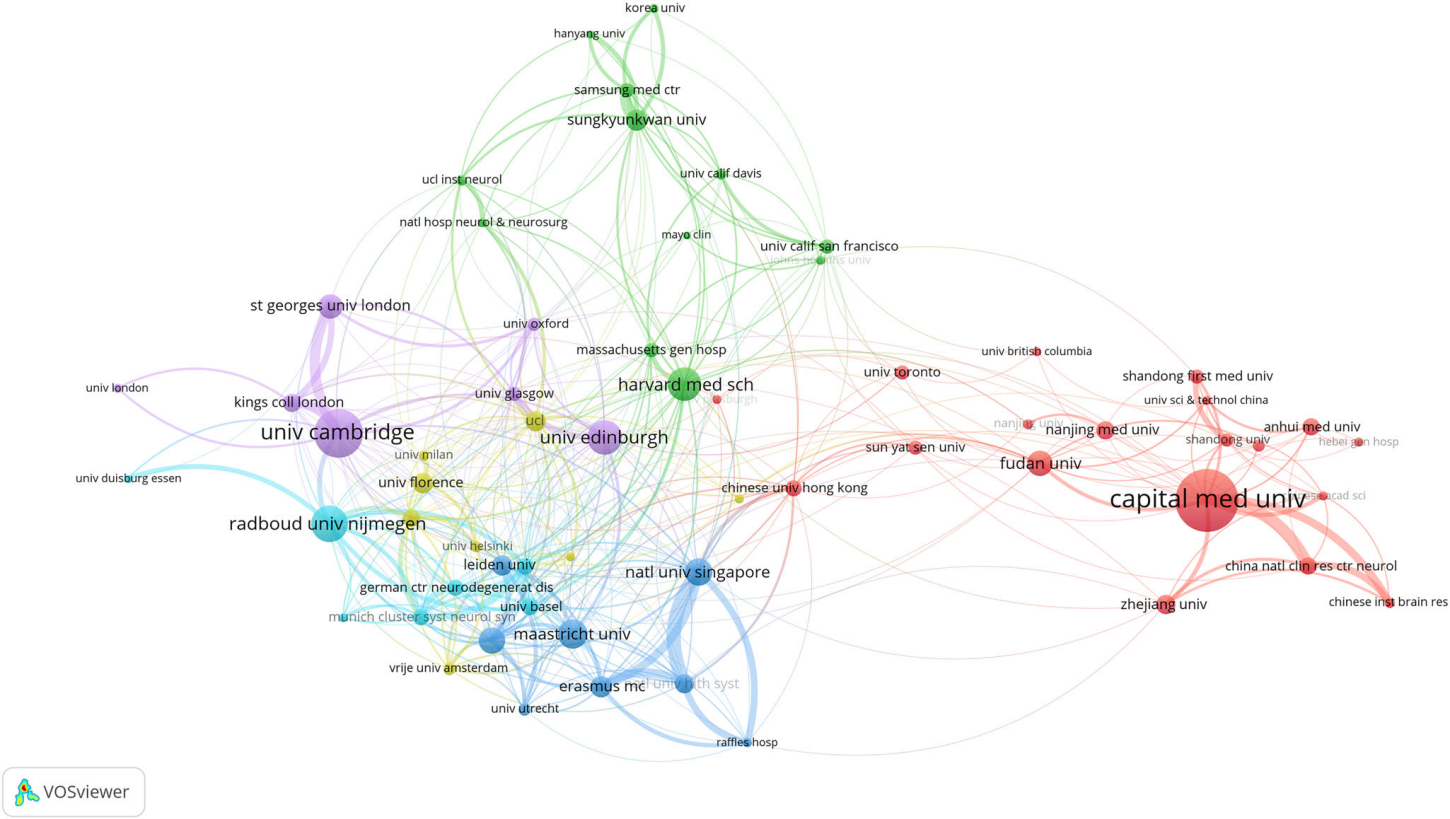
Institutional collaboration network in CSVD‐CI research. The network includes 61 institutions that met the threshold of at least 10 publications. Node size corresponds to the publication count of each institution. Colors represent distinct collaborative clusters identified by VOSviewer's clustering algorithm. Connecting lines indicate co‐authorship, with line thickness proportional to collaboration strength.

The institutional collaboration network provides crucial insights into the organizational structure of CSVD‐CI research. Analysis encompassed 1404 institutions globally, with the network visualization (Figure 5) featuring 61 that met the threshold of 10 publications. Quantitatively, Capital Medical University led in output (75 publications), followed by the University of Cambridge (58 publications) and Radboud University Nijmegen (44 publications). The University of Cambridge demonstrated the strongest academic influence with 2165 total citations.

The network revealed distinct collaborative clusters. The East Asian cluster, led by Capital Medical University and the China National Clinical Research Center for Neurological Diseases, focuses on clinical manifestations and cognitive assessment in Asian populations. The North American‐European Alliance, centered around Harvard Medical School, advances neuroimaging techniques and biomarker development. The European Network, anchored by Radboud University Nijmegen, contributes longitudinal studies on CSVD progression. The UK Collaboration cluster, led by the University of Cambridge, emphasizes population‐based studies and genetic risk factors.

Table 2 identifies institutions with the most pronounced citation bursts in the CSVD‐CI literature from 2001 to 2024, reflecting periods of notable academic influence. King's College London recorded the highest burst strength (8.17), followed by Nanjing Medical University (7.97) and St George's, University of London (6.25). The recent emergence of Nanjing Medical University since 2022 underscores its significant contributions to the field, while Zhejiang University and the University of Basel have also garnered considerable attention. This analysis highlights leading global institutions, presenting potential avenues for international collaboration, academic exchange, and research training.

**TABLE 2:** Institutions with the strongest citation bursts: strength, duration, and publication‐citation profile (2001–2024).

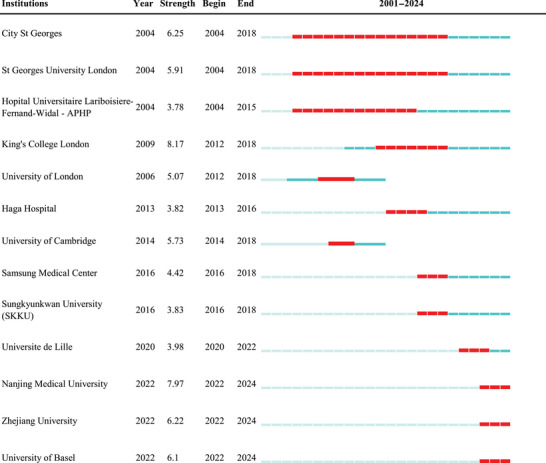	

*Note*: 1. Data source: Web of Science Core Collection (2001–2024), analyzed via CiteSpace software for citation burst detection. 2. Year column definition: The “Year” column indicates the initial year when the citation burst of each institution was first detected in the dataset. 3. Citation burst metrics: “Strength” represents the intensity of citation bursts for each institution; “Begin” and “End” indicate the actual start and end years of the sustained citation burst period. 4. Temporal scope: The 2001–2024 column displays the citation burst trend of each institution over the study period (black bars represent the duration of burst activity). 5. Institutional nomenclature: Abbreviations (e.g., APHP for Assistance Publique–Hôpitaux de Paris) follow the original database labeling conventions.)

#### 3.3.3 Author Cooperation Network

Research in CSVD‐CI represents an evolving field that continues to attract significant scholarly attention. The bibliometric analysis encompassed 1074 papers contributed by 5835 authors. Author collaboration analysis was employed to identify influential researchers and their associated social collaboration networks (Chen 2017).

**FIGURE 6:**
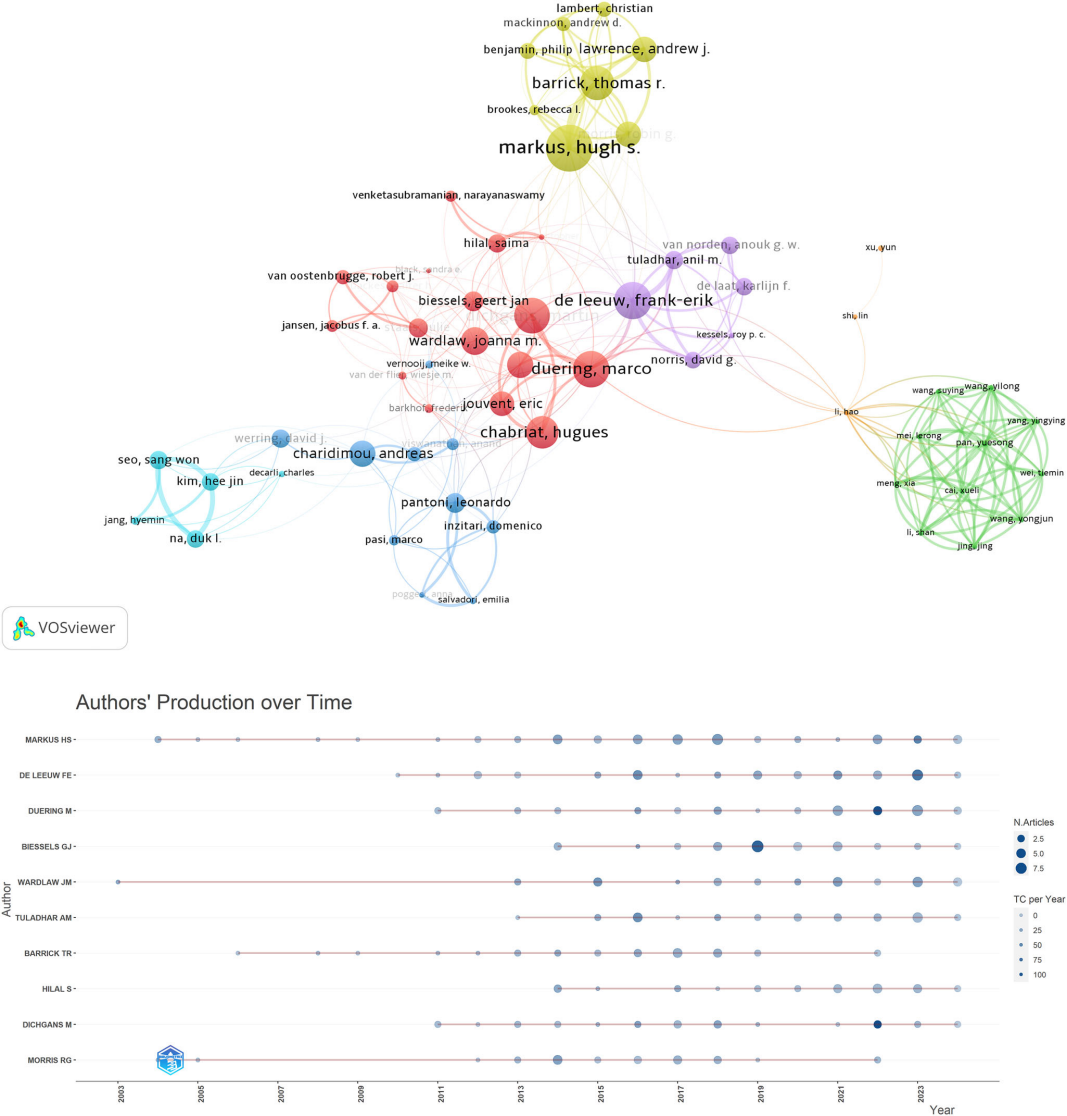
Bibliometric analysis of leading authors in CSVD‐CI research. (A) Co‐authorship network of the 60 most representative authors. Node size corresponds to total citation count, and colors represent distinct collaborative clusters; and (B) temporal trend of annual publication output and citation impact for the top 10 authors. Bubble size indicates the number of publications per year, and color intensity represents the annual citation count.

The author co‐authorship network (Figure 6A) reveals seven major research clusters, with the most highly cited leaders of each cluster being Duering, Marco (1593 citations), Pan, Yuesong (191 citations), Charidimou, Andreas (1089 citations), Markus, Hugh S. (2085 citations), De Leeuw, Frank‐Erik (1607 citations), Seo, Sang Won (729 citations), and Xu, Yun (194 citations), demonstrating diverse leadership across research subdomains.

Complementing the structural analysis, the temporal trends in publication output and citation impact for leading authors are visualized in Figure 6B. The analysis identifies Wardlaw, Joanna M., Markus, Hugh S., and Morris, Robin G. as pioneering researchers, with their foundational work dating to 2003–2004. Quantitative analysis shows that while Wardlaw, Joanna M. ranks fifth in total output (31 publications), she has maintained exceptional recent impact through high citation rates. From 2014 onward, Markus, Hugh S. emerged as the field's most prolific contributor (54 publications), whose scholarly influence peaked in 2023. De Leeuw, Frank‐Erik, ranking second in productivity (91 publications since 2010), demonstrated sustained impact. Furthermore, Duering, Marco and Dichgans, Martin exhibited significant impact in 2022.

The author co‐authorship network reveals seven major research clusters led by highly cited investigators, including Duering, Marco, and Charidimou, Andreas (Figure 6A). Analysis of temporal trends (Figure 6B) identifies Wardlaw, Joanna M., Markus, Hugh S., and Morris, Robin G. as pioneering researchers, with their foundational work dating back to 2003–2004. While Wardlaw, Joanna M. ranks fifth in total output (31 publications), she has maintained exceptional recent impact through high citation rates. From 2014 onward, Markus, Hugh S. emerged as the field's most prolific contributor (54 publications), whose scholarly influence peaked in 2023 with an average of 162.5 citations per year. De Leeuw, Frank‐Erik, ranking second in productivity (91 publications since 2010), demonstrated sustained impact with 75 citations per year in 2023. Furthermore, Duering, Marco and Dichgans, Martin exhibited significant impact in 2022, with 108 and 111 citations per year, respectively.

### 3.4 Co‐Citation Analysis

Co‐citation frequency, defined as the simultaneous citation of two papers within subsequent literature, serves as a fundamental bibliometric measure. Co‐citation analysis has emerged as one of the most effective methodological approaches for examining and mapping scientific knowledge bases (Chen 2017;Small 1973). This analytical technique enables researchers to uncover latent patterns and research trajectories within a given domaint (Chen 2017).

To systematically identify both the core journals and the fundamental knowledge base in the CSVD‐CI field, we employed CiteSpace to conduct comprehensive analyses of author co‐citations, source journal co‐citations, and document co‐citations. This approach provides a robust framework for identifying key contributors, influential journals, and foundational studies that shape the intellectual structure of the field.

#### 3.4.1 Author Co‐Citation Analysis

**FIGURE 7:**
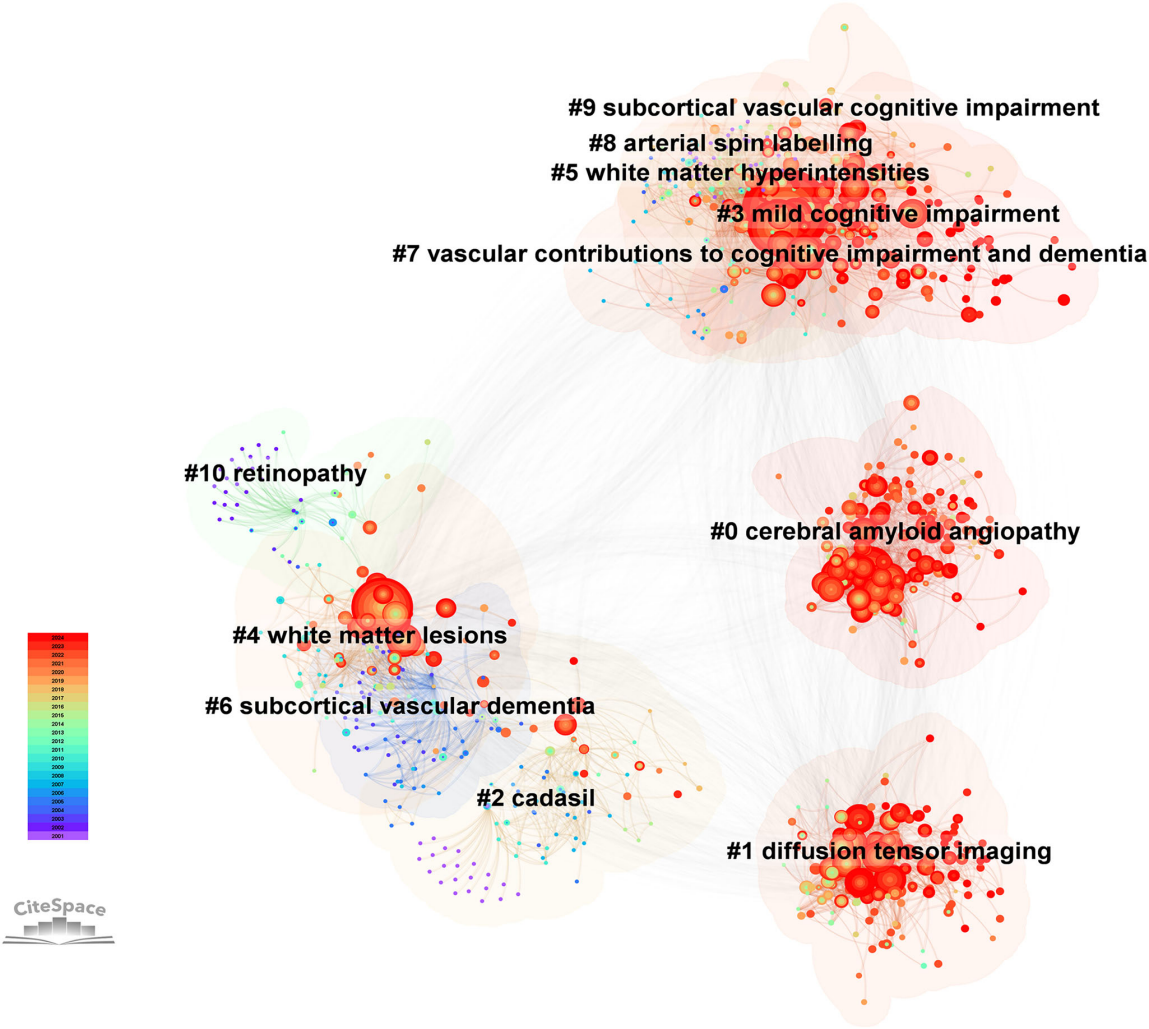
Author co‐citation network clustering. Nodes represent cited authors, with size proportional to co‐citation frequency. Colors denote distinct research clusters, and connecting lines reflect co‐citation relationships. The network consists of 878 nodes and 6141 links, with clusters automatically labeled by the LLR algorithm based on noun phrases from reference titles.

Author co‐citation analysis reveals the intellectual structure of CSVD‐CI research by tracking frequently cited author pairs (Chen 2017; White and Griffith 1981). Figure 7 illustrates the resulting co‐citation network, which comprises 878 nodes and 6141 co‐citation links, with node size representing citation frequency. The analysis identifies 11 distinct thematic clusters, among which four exhibit particularly prominent influence, as indicated by larger node sizes and higher co‐citation frequencies: “#0 cerebral amyloid angiopathy (CAA),” “#1 diffusion tensor imaging (DTI),” “#3 mild CI (MCI),” and “#4 WMLs.”

CAA involves β‐amyloid deposition and presents with cerebral hemorrhage and transient neurological symptoms, often accompanied by progressive cognitive decline (Case et al. 2016; Cozza et al. 2023). DTI detects white matter microstructural damage in mild CI (MCI), correlating with deficits in attention, memory, and executive function (Srisaikaew et al. 2020), and offers a potential tool for early MCI identification (Allen et al. 2019). MCI itself represents a transitional stage between normal aging and dementia, serving as a critical window for early intervention (Zhuang et al. 2021). WMLs, as established neuroimaging markers of CSVD, strongly predict cognitive decline and dementia onset (Hu et al. 2022).

These four themes are highly interconnected: CAA and WMLs represent key vascular pathologies linked to CI, DTI provides the imaging means to detect associated white matter damage, and MCI constitutes the clinical syndrome targeted for early detection and intervention. Together, they form a cohesive framework for understanding CSVD‐related CI, informing strategies for prevention, diagnosis, and treatment.

#### 3.4.2 Literature Co‐Citation Analysis

**FIGURE 8:**
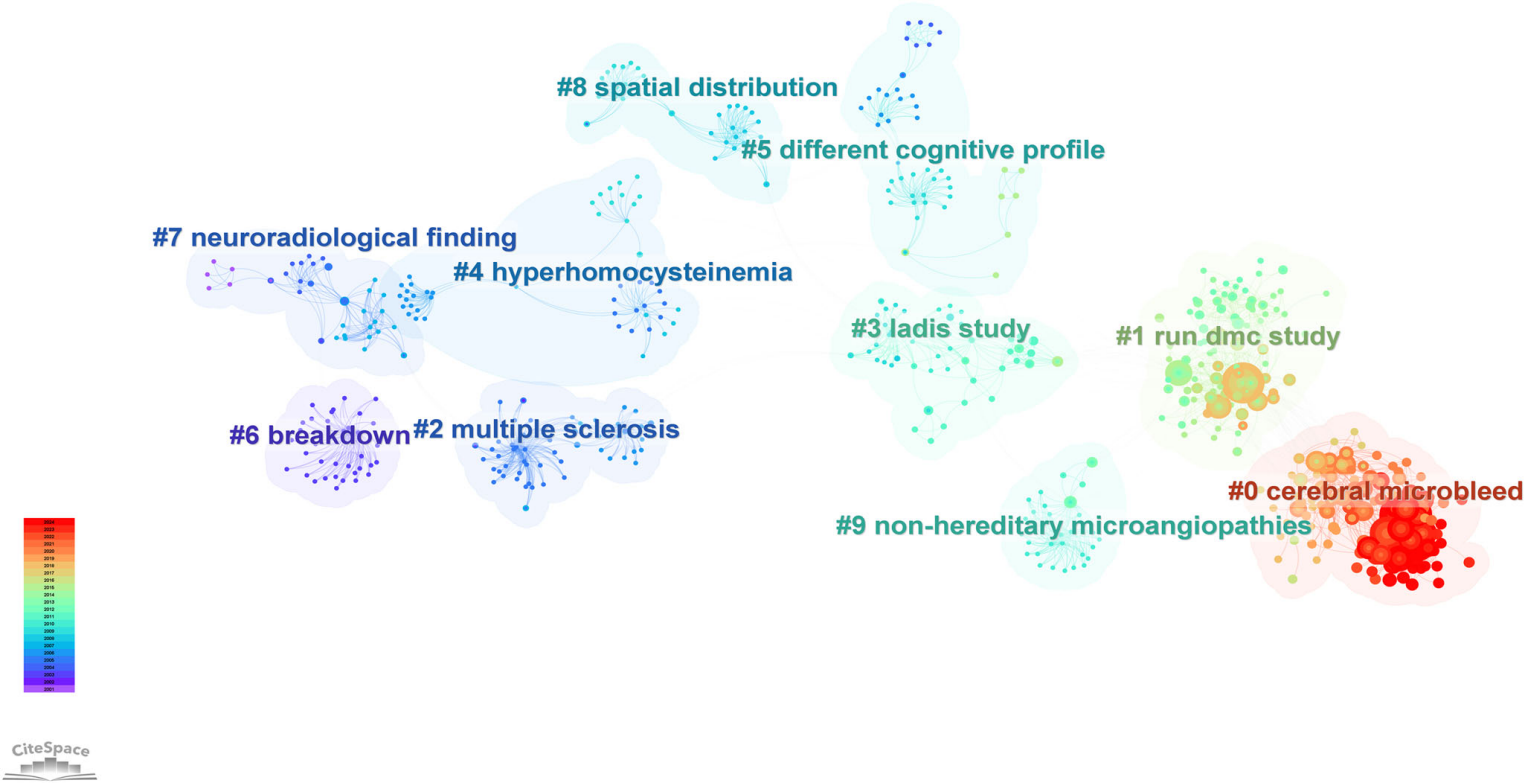
Reference co‐citation network and clustering. Nodes represent cited references, and links represent **co**‐citation relationships. The network is composed of 1110 references and is divided into 51 thematic clusters using the log‐likelihood ratio (LLR) algorithm, with robust structural validity (modularity *Q* = 0.847, mean silhouette *S* = 0.9475). Selected major clusters are labeled.

The reference co‐citation network (Figure 8), fundamental for mapping the knowledge base of a research field (Chen 2017;Small 1973), delineates the intellectual structure of CSVD‐CI research. Analysis of 1110 references identified 51 distinct clusters, with the following ten representing primary research themes: (1) cerebral micro bleeds as neuroimaging markers for dementia risk; (2) the RUN DMC Study on CSVD progression; (3) multiple sclerosis and white matter damage; (4) the LADIS Study on neuroimaging‐cognition correlations; (5) hyperhomocysteinemia in vascular CI pathogenesis; (6) different cognitive profiles showing domain‐specific deterioration; (7) brain function breakdown; (8) comprehensive neuroradiological findings; (9) spatial distribution of cerebral microbleeds; and (10) non‐hereditary microangiopathies. The interconnected nature of these clusters underscores the multifaceted research on CI in CSVD, spanning molecular mechanisms to clinical applications, and provides a comprehensive framework for understanding CSVD‐related cognitive dysfunction.

### 3.5 Keyword Analysis: Research Tendencies and Hotspots

#### 3.5.1 Keywords Co‐Occurrence Analysis

**FIGURE 9:**
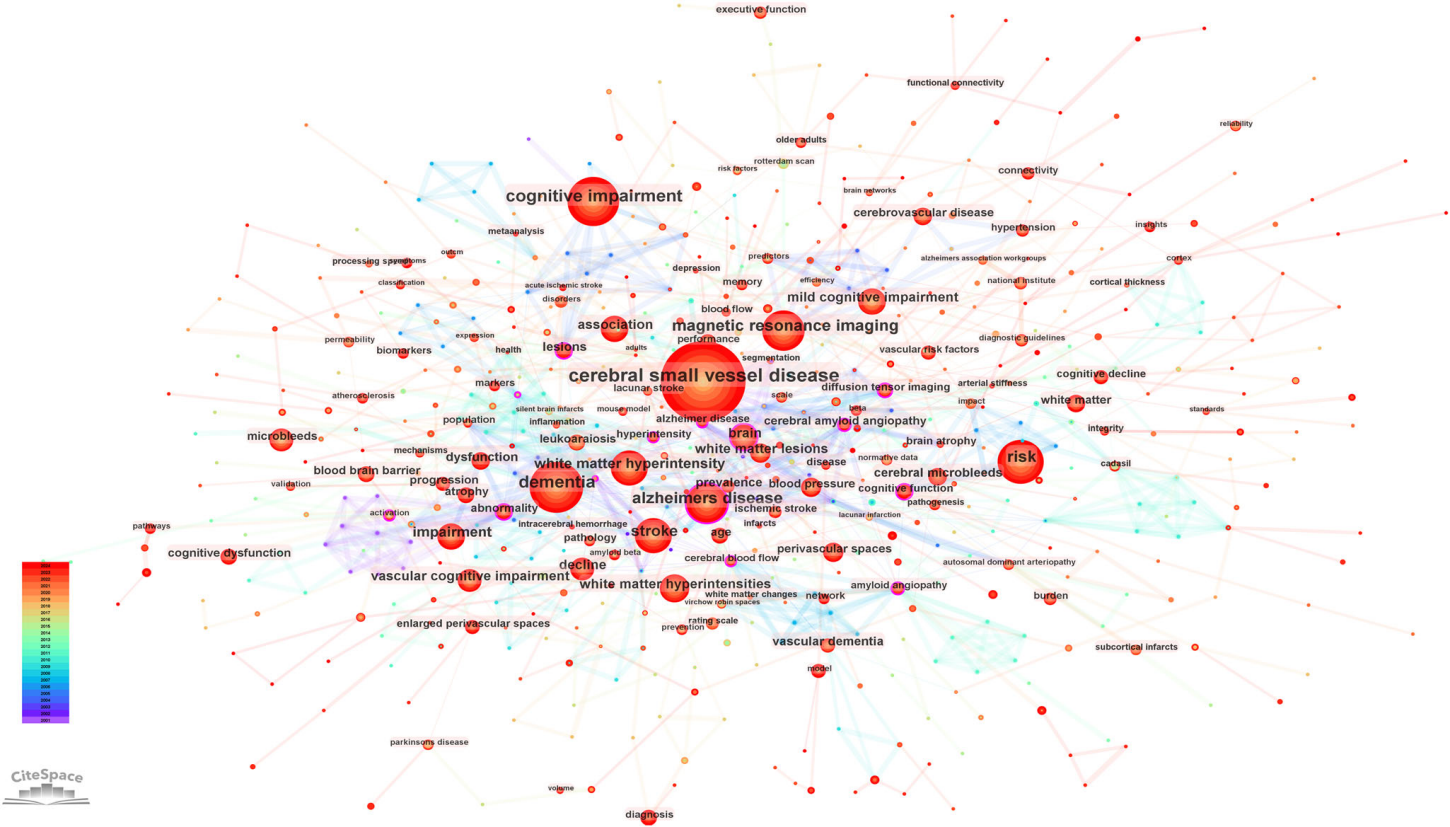
Keyword co‐occurrence network in CSVD‐CI research. The network comprises 576 nodes (keywords) and 1375 links (co‐occurrence relationships). Node size corresponds to the frequency of the keyword, and the lines between nodes represent their co‐occurrence in the literature. The color of the nodes indicates their membership in different thematic clusters.

Keyword co‐occurrence analysis was employed to identify primary research domains in CSVD‐CI by examining the joint presence of terms across publications. The resulting network comprises 576 nodes and 1375 links (Figure 9). Each node represents a keyword, with size proportional to its frequency of occurrence, and connecting lines indicate co‐occurrence relationships within the literature (van Eck and Waltman 2010).

The network exhibits a modularity (Q) value of 0.7096, significantly exceeding the 0.3 threshold (Newman 2006), indicating a well‐defined community structure with loosely coupled clusters. This supports the view that CSVD‐CI research encompasses relatively dispersed yet distinct thematic foci.

As detailed in Table 3, the most frequent keywords define the core research areas: “CSVD” (frequency = 766), “CI” (frequency = 357), “dementia” (frequency = 348), “magnetic resonance imaging” (frequency = 276), and “risk” (frequency = 274). Concurrently, keywords with high betweenness centrality—including “abnormality” (centrality = 0.19), “amyloid angiopathy” (centrality = 0.15), and “lesions” (centrality = 0.14)—function as pivotal hubs connecting multiple research themes. In CiteSpace, centrality measures the degree to which a node lies on the shortest paths between others, with values above 0.1 considered highly influential. These high‐centrality keywords therefore play critical roles in bridging different research topics and facilitating the integration of knowledge across the CSVD‐CI domain.

**TABLE 3:** Top effective keywords in the field of CSVD‐CI research, ranked by frequency (>100) and centrality (>0.1).

Keywords	Frequency	Year	Centrality		Year
Cerebral small vessel disease	766	2001	Abnormality	0.19	2001
Cognitive impairment	357	2001	Amyloid angiopathy	0.15	2005
Dementia	348	2002	Lesions	0.14	2003
Magnetic resonance imaging	276	2001	Cognitive function	0.13	2005
Risk	274	2006	Diffusion tensor imaging	0.13	2006
Alzheimers disease	258	2002	Alzheimer disease	0.13	2003
Stroke	179	2002	Cerebral blood flow	0.13	2002
White matter hyperintensity	172	2003	Activation	0.13	2012
Impairment	128	2005	Follow up	0.13	2004
Brain	118	2001	Alzheimers disease	0.12	2002
Association	117	2009	Brain infarction	0.12	2002
White matter hyperintensities	116	2012	Brain	0.11	2001
White matter lesions	113	2001	Cerebral amyloid angiopathy	0.11	2003
Mild cognitive impairment	108	2004	Disease	0.1	2004
Decline	103	2002	Hyperintensity	0.1	2004
Vascular cognitive impairment	100	2009	Dementia	0.1	2002

*Note*: 1. Data source: Web of Science Core Collection (2001–2024), analyzed using CiteSpace software for keyword co‐occurrence and betweenness centrality calculation. 2. Selection criteria: Keywords were filtered by frequency (>100 occurrences) and betweenness centrality (>0.1), representing the most impactful terms in the CSVD‐CI research field. 3. Column definitions: ‐ “Frequency”: Total occurrence count of each keyword in the dataset; ‐ “Year” (left): The first year the keyword appeared in CSVD‐CI‐related publications; ‐ “Centrality”: Betweenness centrality value (CiteSpace metric), with values >0.1 indicating high influence as a research topic hub; ‐ “Year” (right): The first year the high‐centrality keyword emerged as a pivotal research term. 4. Abbreviation: CSVD‐CI = Cerebral Small Vessel Disease‐Related Cognitive Impairment.

#### 3.5.2 Keyword Cluster Analysis

**FIGURE 10:**
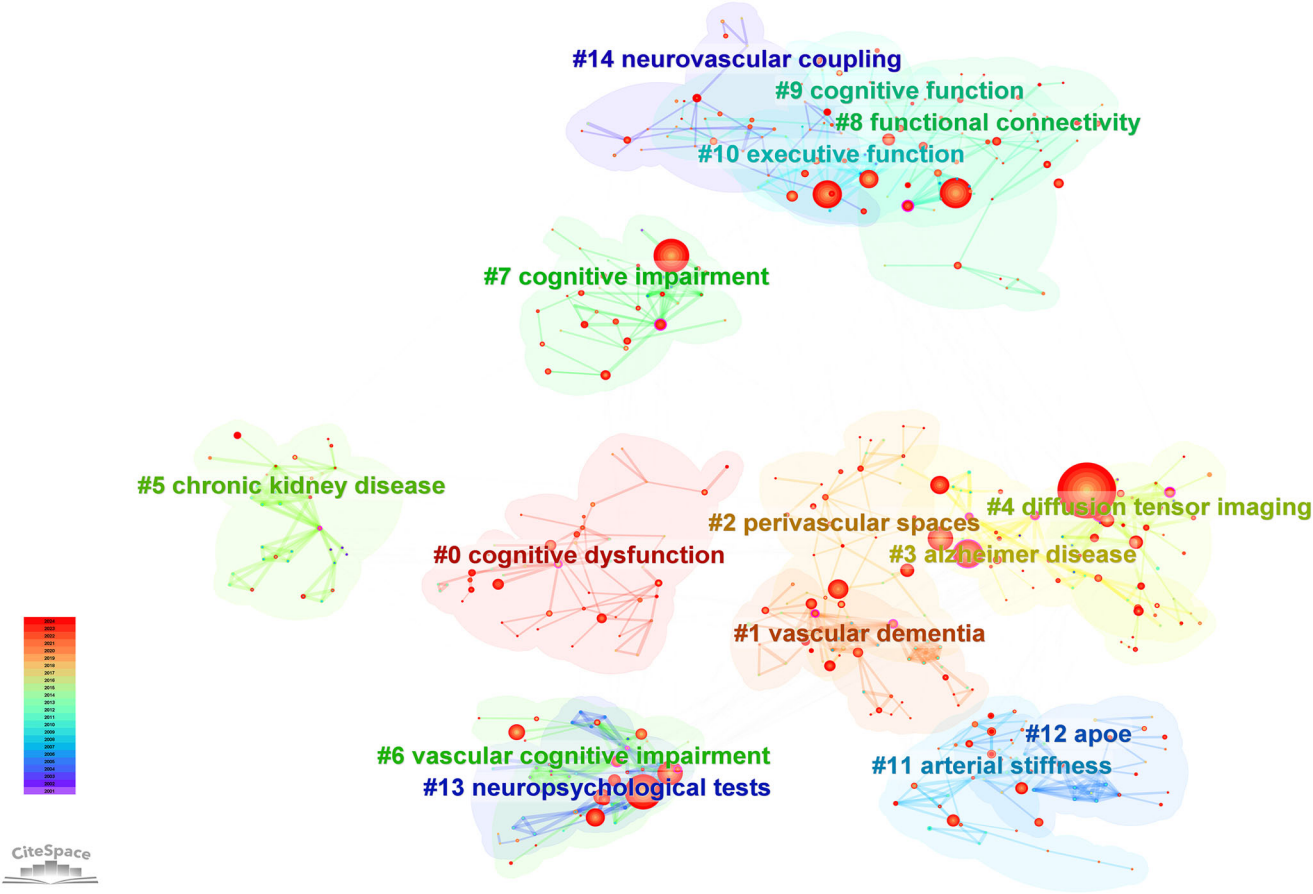
Clustered keyword co‐occurrence network. The network is partitioned into distinct thematic clusters using the LLR algorithm in CiteSpace, with each cluster automatically labeled by its most characteristic keyword. Cluster numbers (#0, #1, etc.) indicate relative size, with #0 being the largest. A detailed overview of all clusters, including their size, silhouette value, and top keywords, is provided in Table 4.

Keyword cluster analysis was performed using CiteSpace to elucidate the thematic architecture of CSVD‐CI research. Keywords were categorized into clusters based on co‐occurrence patterns, with each cluster automatically labeled using the LLR algorithm (Chen 2006, 2017). The analysis identified 15 distinct clusters with robust structural validity (mean silhouette value = 0.88), visually presented in Figure 10 and detailed in Table 4.

**TABLE 4:** Knowledge clusters based on keywords co‐occurrence.

Rank	Size	Mean contour value	Peak year	Top 5 high‐frequency keywords(The first keyword serves as the label for each cluster)
0	48	0.838	2018	Cognitive dysfunction (13.61, 0.001); oxidative stress (12.45, 0.001); cortical microinfarcts (8.72, 0.005); apolipoprotein e (8.51, 0.005); mild cognitive impairment (8.39, 0.005)
1	43	0.945	2013	Vascular dementia (42.09, 1.0E‐4); amyloid angiopathy (12.89, 0.001); pressure (11.76, 0.001); amplitude of low‐frequency fluctuation (11.76, 0.001); rating scale (11.14, 0.001)
2	38	0.758	2018	Perivascular spaces (21.85, 1.0E‐4); white matter hyperintensities (19.28, 1.0E‐4); machine learning (9.34, 0.005); blood flow (8.21, 0.005); single‐photon emission computed tomography (7.83, 0.01)
3	38	0.891	2010	Alzheimer disease (19.28, 1.0E‐4); cerebral amyloid angiopathy (11.95, 0.001); cadasil (11.38, 0.001); cerebrovascular disorders (11.37, 0.001); white matter hyperintensity (10.69, 0.005)
4	38	0.894	2009	Diffusion tensor imaging (39.97, 1.0E‐4); cognition (19.3, 1.0E‐4); white matter lesions (13.67, 0.001); small vessel disease (13.42, 0.001); cerebral small vessel disease (11.42, 0.001)
5	35	0.895	2011	Chronic kidney disease (27.86, 1.0E‐4); health (15.17, 1.0E‐4); glomerular filtration rate (8.88, 0.005); stroke‐prone renovascular hypertensive rats (8.18, 0.005); synthase (8.18, 0.005)
6	34	0.894	2008	Vascular cognitive impairment (33.82, 1.0E‐4); intracerebral hemorrhage (10.29, 0.005); abnormality (8.84, 0.005); stroke (6.92, 0.01); silent brain infarcts (5.95, 0.05)
7	34	0.882	2013	Cognitive impairment (25.79, 1.0E‐4); progression (11.07, 0.001); acute ischemic stroke (11.07, 0.001); depression (8.15, 0.005); cognitive function (7.15, 0.01)
8	32	0.917	2020	Functional connectivity (23.69, 1.0E‐4); predictive modeling (14.56, 0.001); rich‐club (14.56, 0.001); lasso regression (14.56, 0.001); graph theory (11.53, 0.001)
9	32	0.794	2016	Cognitive function (16.33, 1.0E‐4); diffusion tensor imaging (11.31, 0.001); risk (10.2, 0.005); vascular cognitive impairment (8.74, 0.005); white matter lesions (7.24, 0.01)
10	31	0.861	2011	Executive function (21.65, 1.0E‐4); mild cognitive impairment (19.33, 1.0E‐4); cerebrovascular disease (17.51, 1.0E‐4); magnetic resonance imaging (15.7, 1.0E‐4); executive functions (9.91, 0.005)
11	30	0.942	2015	Arterial stiffness (11.59, 0.001); cerebrovascular reactivity (8.11, 0.005); pulse wave velocity (7.97, 0.005); blood flow velocity (7.32, 0.01); brain infarction (7.32, 0.01)
12	30	0.9	2015	Apoe (15.18, 1.0E‐4); alopecia (15.18, 1.0E‐4); mutation (11.41, 0.001); htra1 (9.73, 0.005); surrogate marker (9.73, 0.005)
13	29	0.958	2009	Neuropsychological tests (14.57, 0.001); rs‐fmri (9.71, 0.005); cognition impairment (9.71, 0.005); cognition (8.14, 0.005); neurovascular coupling (7.99, 0.005)
14	24	0.77	2018	Neurovascular coupling (14.43, 0.001); positron emission tomography (13.34, 0.001); randomized controlled trial (13.34, 0.001); tspo (18 kda translocator protein) (8.55, 0.005); retinal vessel density (8.55, 0.005)

*Note*: 1. Data source: Web of Science Core Collection (2001–2024), analyzed via CiteSpace software for keyword co‐occurrence clustering (cluster analysis parameters: pruning = pathfinder, slicing = 2001–2024). 2. Column definitions: ‐ “Rank”: Cluster ranking by size (0 represents the largest cluster); ‐ “Size”: Number of nodes (keywords) included in each cluster, indicating relative cluster scale; ‐ “Mean contour value”: Silhouette coefficient (range: 0–1), reflecting the homogeneity and cohesion of the cluster (values >0.5 indicate high cluster validity); ‐ “Peak year”: The year when the cluster reached its highest research activity in the CSVD‐CI field; ‐ “Top 5 high‐frequency keywords”: The five most frequent keywords in each cluster; the first keyword serves as the cluster label, with parentheses indicating keyword frequency and statistical significance (p‐value).

These clusters, spanning key research areas from #0 cognitive dysfunction to #14 neurovascular coupling, were synthesized into broader thematic domains for interpretation. The cognitive domain (clusters #0, #6, #7) focuses on the progression of cognitive deficits, incorporating mechanisms like oxidative stress. The dementia spectrum (clusters #1, #3) highlights the intersection of vascular and alzheimer's pathologies. Neuroimaging markers (clusters #2, #4) underscore perivascular spaces and DTI as crucial disease indicators. Functional aspects (clusters #8, #9, #10) explore CSVD's impact on brain connectivity and executive control. Finally, vascular mechanisms (clusters #11, #14) investigate arterial stiffness and neurovascular coupling.

This synthesis demonstrates the multifaceted nature of CSVD‐CI research, connecting vascular pathology to cognitive outcomes through advanced imaging and functional assessment.

#### 3.5.3 Keywords Burst Detection Analysis

Burst detection analysis was employed to observe rapidly growing topics by identifying keywords that showed sudden increases in frequency over short periods, thereby understanding topic evolution in recent years (Chen 2006, 2017). Through the construction of keyword co‐occurrence networks in CSVD‐CI research from 2001 to 2024, burst keywords were detected as shown in Table 5. These 22 keywords received the most attention from 2001 to 2024.

**TABLE 5:** Keywords with the strongest citation bursts.

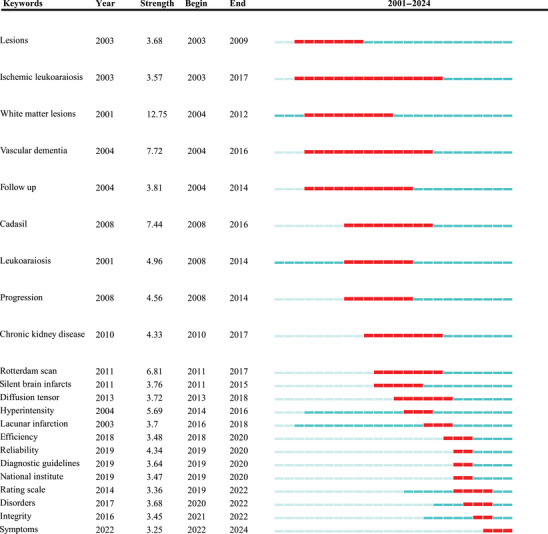	

*Note*: 1. Data source: Web of Science Core Collection (2001–2024), analyzed via CiteSpace software for citation burst detection of keywords in the CSVD‐CI research field (22 burst keywords identified in total). 2. Column definitions: ‐ “Year”: The initial year when the citation burst of each keyword was first detected in the dataset; ‐ “Strength”: Intensity value of the keyword’s citation burst, reflecting the magnitude of research attention received; ‐ “Begin”/“End”: The actual start and end years of the sustained citation burst period for each keyword; ‐ “2001–2024”: The black bar represents the temporal duration of the citation burst for each keyword over the study period. 3. Abbreviation: CADASIL (Cadasil in table) = Cerebral Autosomal Dominant Arteriopathy with Subcortical Infarcts and Leukoencephalopathy.

Bibliometric analysis of CSVD‐CI research from 2001 to 2024 reveals several distinctive trends in keyword frequency and evolution. Most notably, “WMLs” has consistently emerged as a predominant research focus, with the highest strength index (12.75), demonstrating its pivotal role in CSVD‐CI research, particularly during the period of 2004–2012. Additionally, keywords such as “vascular dementia” and “leukoaraiosis” exhibited significant prominence across various time periods, highlighting the crucial relationship between CSVD and CI.

Recent years have witnessed the increasing application of advanced imaging techniques, particularly “diffusion tensor” imaging, providing novel perspectives for investigating the microstructural characteristics of CSVD‐CI. Furthermore, the emergence of keywords such as “efficiency,” “reliability,” “diagnostic guidelines,” and “symptoms” between 2018 and 2024 indicates ongoing exploration in optimizing diagnostic and therapeutic approaches, with particular emphasis on diagnostic criteria, reliability assessment, and symptomatology. The overall trajectory demonstrates a progressive evolution from pathophysiological mechanisms to sophisticated diagnostic and clinical applications. These trends underscore the continuous advancement in CSVD‐CI research, with emerging technologies and methodologies providing new impetus and directions for future development in this field.

## 4 Discussion

This bibliometric analysis provides a comprehensive overview of the evolution and current state of research on CSVD‐CI from 2001 to 2024. The findings reveal several significant patterns and trends, including the evolution of research focus, emerging research priorities, research collaboration patterns, and an increasing emphasis on identifying risk factors and advancing diagnostic and therapeutic approaches. Below, we discuss the key findings and their implications within the context of existing literature.

### 4.1 Evolution of Research Focus

Our bibliometric analysis clearly delineates the evolution of CSVD‐CI research through three phases, each with a characteristic publication growth slope driven by distinct scientific and clinical developments.

The foundational phase (2001–2010) was marked by a shallow growth curve, consistent with a field in its infancy, building the essential epidemiological and pathological groundwork.

The subsequent developmental phase (2011–2018) exhibited a steep upward slope in publications. This acceleration was not merely quantitative but qualitative, fueled by the critical maturation of neuroimaging standards and the formation of large international cohorts. The establishment of the STRIVE criteria was a pivotal event that provided the necessary methodological unity for the field to expand rapidly and collaboratively.

Finally, the acceleration phase (2019–2024) maintained a high velocity of output on an increasingly large base. The growth in this phase reflects the field's expansion into novel mechanistic domains (e.g., neurovascular unit dysfunction, glymphatic system) and a intensified focus on clinical translation, including drug trials and multi‐modal biomarker discovery. The sustained high level of activity indicates that CSVD‐CI has solidified its position as a major, dynamic research frontier in neuroscience.

### 4.2 Emerging Research Priorities

Our keyword analysis has identified several critical research priorities within the field of CSVD‐CI. Firstly, pathophysiological mechanisms are gaining prominence, with “WMLs” exhibiting a high burst strength of 12.75, underscoring their pivotal role in CSVD pathology. There is increasing focus on neurovascular coupling and BBB dysfunction (Che et al. 2024; Li et al. 2024; Wang et al. 2023; Yang and Webb 2023), which are emerging as key mechanistic insights, alongside growing attention to oxidative stress and neuroinflammation as primary pathogenic factors (Kancheva et al. 2024). Secondly, neuroimaging biomarkers are evolving from conventional structural imaging to more advanced techniques, such as DTI, with the integration of machine learning approaches for enhanced imaging analysis (Warren and Moustafa 2023). The emphasis is shifting toward multi‐modal imaging to provide a more comprehensive characterization of the disease. Lastly, clinical translation is becoming a significant area of interest, marked by the recent emergence of keywords related to diagnostic guidelines and reliability between 2018 and 2024. This shift is further evidenced by a growing focus on the standardization of assessment tools and trial methodologies in the wake of landmark studies such as the sprint‐mind trial, which has shaped an international perspective on clinical trial design for vascular CI (Elahi et al. 2023).

### 4.3 Research Collaboration Patterns

The analysis reveals distinct geographical clusters of research activity, highlighting robust collaboration networks centered around North American institutions (led by the United States), European research centers (particularly in the Netherlands and the United Kingdom), and Asian institutions (mainly in China). Despite these established networks, there is significant potential for enhanced international collaboration, which could substantially accelerate knowledge exchange and research progress in the field. Strengthening these collaborations could lead to more comprehensive studies and a better understanding of the global impact of CSVD‐CI.

### 4.4 Risk Factors and Emerging Focus on Sleep Disorders

In addition to traditional vascular risk factors such as hypertension, diabetes, and hyperlipidemia, emerging evidence highlights the critical role of sleep disorders in CSVD‐CI. Sleep disturbances, including insomnia and sleep apnea, have been associated with an increased burden of WMH, impaired executive function, and accelerated cognitive decline (Hosoya et al. 2023; Kaneshwaran et al. 2019; Kocevska et al. 2019). Sleep fragmentation and reduced sleep efficiency may exacerbate vascular dysfunction and neuroinflammation, further contributing to the progression of CSVD (Lim and Pack 2014; Zolotoff et al. 2021). Addressing sleep disorders in CSVD patients represents a novel therapeutic target that could mitigate cognitive decline and improve overall outcomes.

### 4.5 Diagnostic and Therapeutic Implications

The development of standardized diagnostic criteria, such as the standards for reporting vascular changes on neuroimaging (STRIVE), has significantly advanced the field by enabling consistent evaluation of CSVD burden. The total CSVD score, a semi‐quantitative approach combining multiple lesion types, has proven to be a reliable method for assessing disease severity and predicting cognitive outcomes (Huijts et al. 2013; Staals et al. 2015). Furthermore, brain network analyses using graph theory have emerged as sensitive markers of cognitive dysfunction, offering potential as surrogate biomarkers for CSVD‐CI (Da et al. 2023; Lawrence et al. 2014).

Despite these advances, no specific treatment has been identified to effectively halt or reverse cognitive decline in CSVD‐CI. Current therapeutic strategies focus on managing vascular risk factors and optimizing lifestyle interventions. Future research should prioritize the development of targeted therapies that address the underlying pathophysiological mechanisms, such as neuroinflammation and BBB dysfunction, as well as interventions aimed at improving sleep quality in CSVD patients.

### 4.6 Methodological Considerations

In conducting this bibliometric analysis, we made a deliberate methodological decision to focus exclusively on original research articles (Articles) while excluding review‐type publications. This approach was guided by several core principles of bibliometric science. First, as our primary aim was to map the trajectory of primary knowledge production in the field of CSVD‐CI, the inclusion of reviews—which synthesize existing knowledge rather than report new findings—could obscure the genuine evolution of research fronts and themes (Cooper and Koenka 2012; Marks et al. 2013; Robinson et al. 2014). Second, reviews often function as “super‐nodes” in citation networks due to their disproportionately high citation rates and extensive reference lists. Their inclusion could potentially distort co‐citation and keyword co‐occurrence networks, overshadowing the nuanced relationships between original research clusters (He et al. 2025; Lee et al. 2025). Furthermore, the classification of “review” is inconsistent across journals and databases; restricting our analysis to Articles enhanced the methodological consistency, reproducibility, and cross‐journal comparability of our findings (He et al. 2025; Lee et al. 2025). We acknowledge that this choice means our analysis does not capture the valuable synthesizing function of review articles. However, we maintain that this focused approach provides a clearer and more accurate representation of the development of primary research in CSVD‐CI, and we have ensured full transparency by stating this limitation explicitly.

### 4.7 Limitations and Future Directions

This study has several limitations. First, the bibliometric analysis was restricted to publications indexed in the WoS database, which may have excluded relevant studies from other sources. Second, the inclusion of only English‐language articles may have introduced language bias. Third, citation lag may have led to the underrepresentation of recent publications in the analysis. Fourth, an additional potential limitation stems from our literature selection criteria. To accurately track the trajectory of primary research output, we focused our analysis on original research articles and did not incorporate review literature. While this methodological choice aligns with standard bibliometric practice and helps avoid biases in network analysis, it also means our analysis does not capture the context of knowledge integration and systematization as reflected in review articles.

Future research should focus on longitudinal studies to better understand the progression of CSVD‐CI and its relationship with cognitive decline. Additionally, integrating advanced neuroimaging techniques with molecular biomarkers could enhance early detection and risk stratification. Investigating the interplay between sleep disorders and vascular risk factors in CSVD patients represents another promising avenue for research. Furthermore, more studies are needed to explore the interaction between CSVD and other neurodegenerative diseases, such as alzheimer's disease, to better understand their combined impact on cognitive function. Finally, a dedicated bibliometric analysis of review literature could be conducted in the future to complement our findings by delineating the landscape of knowledge integration in this field.

## 5 Conclusion

This bibliometric analysis highlights the rapid growth and evolving focus of CSVD‐CI research over the past two decades. Advances in neuroimaging have significantly deepened our understanding of the pathophysiological mechanisms underlying CSVD‐CI, particularly the role of structural and functional brain network disruptions. Emerging evidence underscores the importance of addressing modifiable risk factors, such as sleep disorders, to mitigate cognitive decline in CSVD patients. Despite significant progress, many challenges remain, including the need for standardized diagnostic criteria, effective therapeutic interventions, and a deeper understanding of disease progression. Future research should prioritize the integration of multi‐modal imaging and molecular biomarkers, as well as the development of targeted therapies that address both vascular and non‐vascular contributors to CI. Addressing these gaps will be critical for improving outcomes and reducing the global burden of CSVD‐CI.

## Author Contributions

**Kuihua Wang**: conceptualization, investigation, formal analysis, data curation, writing – original draft, funding acquisition. **Xiaohui Ji**: data curation, investigation, formal analysis, writing – original draft, software. **Hui Li**: investigation, validation. **Xiaoyang Wang**: software, investigation. **Xiaoyue Jin**: software, investigation. **YanJun Lin**: data curation, investigation. **Qiao Wang**: data curation, investigation. **Haizhen Xu**: data curation, investigation. **JianxinYe**: investigation, validation. **Xiaoping Cui**: conceptualization, methodology, project pdministration, writing – review and editing. **Yonghui Liang**: conceptualization, methodology, project administration, writing – review and editing, funding acquisition.

## Funding

This work was supported by National Natural Science Foundation of China(Grant No. 82374528), International Cooperation Project of Fujian Provincial Science and Technology Program (Grant No. 2025I0042), the Fujian Provincial Science and Technology Innovation Platform Construction Project (Grant No. 2022Y2017), and the Institutional Research Fund of the 900th Hospital (Grant No. 2022MS16).

## Consent

The authors have nothing to report

## Conflicts of Interest

The authors declare no conflicts of interest.

## Supporting information




**Supplementary Material**: brb371167‐sup‐0001‐SuppMat.xls

## Data Availability

The original bibliometric data used in this study were sourced from the WoS core collection, a publicly available database. The specific search strategy and data processing steps are detailed in the methods section. The analyzed datasets and custom scripts generated during the study are available from the corresponding author upon reasonable request.
